# Fast and precise inference on diffusivity in interacting particle systems

**DOI:** 10.1007/s00285-023-01902-y

**Published:** 2023-03-29

**Authors:** Gustav Lindwall, Philip Gerlee

**Affiliations:** Chalmers tvärgata 3, 412 58 Gothenburg, Sweden

**Keywords:** Interacting particle systems, Glioblastoma, Agent based modelling, Stochastic processes, Stochastic differential equations, Diffusion, Bayesian inference, 92-10, 62F15, 60J60

## Abstract

Particle systems made up of interacting agents is a popular model used in a vast array of applications, not the least in biology where the agents can represent everything from single cells to animals in a herd. Usually, the particles are assumed to undergo some type of random movements, and a popular way to model this is by using Brownian motion. The magnitude of random motion is often quantified using mean squared displacement, which provides a simple estimate of the diffusion coefficient. However, this method often fails when data is sparse or interactions between agents frequent. In order to address this, we derive a conjugate relationship in the diffusion term for large interacting particle systems undergoing isotropic diffusion, giving us an efficient inference method. The method accurately accounts for emerging effects such as anomalous diffusion stemming from mechanical interactions. We apply our method to an agent-based model with a large number of interacting particles, and the results are contrasted with a naive mean square displacement-based approach. We find a significant improvement in performance when using the higher-order method over the naive approach. This method can be applied to any system where agents undergo Brownian motion and will lead to improved estimates of diffusion coefficients compared to existing methods.

**Supplementary Information:**

The online version contains supplementary material available at 10.1007/s00285-023-01902-y.

## Introduction

In many areas of the applied sciences, stochastic differential equations (SDE:s) are a popular and well-studied model framework for modelling processes undergoing both deterministic and random dynamics. Examples of application areas are physics (Van Kampen 1992), chemistry (Van Kampen 1992), biology (Lewis et al. 2013), finance (Shreve 2004) and control theory (Stengel 1986). The application in mind for this paper is models of in vitro cell migration, with the location of a cell at time *t* being denoted as $\textbf{x}(t)$. In its most general form, an *N*-dimensional system of Itô SDE:s is given by the equation

1.1 $$\begin{aligned} \textrm{d}\textbf{x}(t) = \mu (\textbf{x}(t),t)\textrm{d}t + \sigma (\textbf{x}(t),t)\textrm{d}W(t) \end{aligned}$$

where $\mu :\textbf{R}^N\mapsto \textbf{R}^N$ is the drift function, $\sigma$ is an $N\times M$ diffusion matrix and *W*(*t*) is an $M\times N$ standard Wiener process. For convergence and well-posedness, $\mu$ and $\sigma$ has to satisfy a set of standard Lipschitz requirements (Klebaner 2012). This framework allows for a large number of natural phenomena to be modelled and studied in a relatively compact way, and is intrinsically linked to the macroscopic phenomena of diffusion of gases and liquids (Krapivsky et al. 2010), where the drift term corresponds to both external forces and intra-particle interaction.

Regression of such models to fit observed data is an active field of research among both mathematicians (Iacus 2009) and members of the application communities, e.g. in control theory, stochastic differential equations are usually studied in the context of state space models (Schön and Lindsten 2015). In the most basic of cases, expressions for parameter estimates can be found exactly, such as in the seminal Ornstein-Uhlenbeck process often used as a toy example in physics. In one dimension it is expressed as

$$\begin{aligned} \textrm{d}x(t) = -\alpha x(t)\textrm{d}t + \sigma \textrm{d}W(t). \end{aligned}$$

For this equation, we have that the transition probability from a state *x*(*s*) to a future state *x*(*t*) at time $t>s$ follows a normal distribution with time-dependent mean and variance,

1.2 $$\begin{aligned} p(x(t)|x(s))=\sqrt{\frac{\alpha }{\pi \sigma ^2(1-e^{-2\alpha (t-s)})}}\exp \Big (-\frac{\alpha }{\sigma ^2}\frac{(x(t)-x(s)e^{-\alpha (t-s)})^2}{1-e^{-2\alpha (t-s)}}\Big ),\nonumber \\ \end{aligned}$$

and as such a maximum likelihood estimate is readily available for $\alpha$ and $\sigma$ given observed data. For most real-world applications such simple models constitute important building blocks and learning tools, but are generally insufficient to accurately describe or forecast a real-world system. Furthermore, an expression as elegant as (1.2) is impossible to find for almost all models, and the inference must be carried out using some sort of approximation. Approximations can be carried out in a multitude of ways. For example, one might opt for a likelihood-free approach such as Approximate Bayesian Computation (Picchini 2014), or try to simplify the terms of the equation, hopefully resulting in a tractable expression. An example of such a method is local linearisation of the SDE (Shoji and Ozaki 1998).

### Mean square displacement and induced sub-diffusivity

**Fig. 1:**
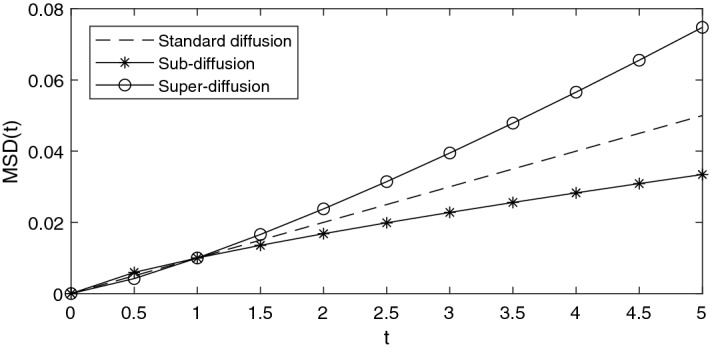
Typical illustration of anomalous diffusion as compared to standard diffusion. In the standard case, corresponding to Brownian motion, the MSD increases linearly with the diffusion coefficient *D*

The estimation of diffusion rates in interacting particle systems have long been a key component in mathematical biology (Swanson et al. 2000). Through the application of Itô calculus, one can find a relationship between individual-based models and partial differential equations (PDEs) describing the population on a macroscopic level (Oelschläger 1989). The archetypical PDE is given as

1.3 $$\begin{aligned} \partial _t u(\textbf{x},t) = D\Delta u(\textbf{x},t) + (N-1)u(\textbf{x},t)\nabla \cdot (V*u(\textbf{x},t)). \end{aligned}$$

Here *u* is the population density, *N* is the number of particles, *D* is the diffusion coefficient and *V* is a function determined by the pairwise interactions (Bruna et al. 2017). The standard procedure of estimating the diffusion coefficient in this equation is to evaluate the mean square displacement (MSD) of individuals in the population, and drawing conclusions from there. For example, in Kwon et al. (2019) Kwon et al. perform a comprehensive study of the diffusivity of migrating lung cancer cells using MSD as the main tool for inference. Likewise, in Wu et al. (2014) Wu et al. reach the conclusion that Gaussian random walks are insufficient to model 3D cell migration in the presence of a complex extra-cellular matrix. One can also measure diffusivity by tackling the PDE (1.3) directly; see for example (Swanson 2008) for an approach applying equations similar to (1.3) directly to in vitro data.

High particle density and interaction forces on otherwise Brownian particles leads to a deviation from their standard diffusive behavior, a phenomena known as induced sub-diffusivity. This problem has been studied by physicists for the last few decades, see for example (Spiechowicz and Łuczka 2017) and (Ledesma-Durán et al. 2021). Thus, just considering the MSD of our particle system will not suffice to draw conclusions regarding the diffusion coefficient in the cases of dense, highly correlated particles. In this paper we present a solution to the problem of estimating diffusivity if a mechanistic model of cell-to-cell interaction is available in the form of an SDE system.

### Our contributions

In this paper, we will cover a Bayesian conjugacy for certain types of interacting particle systems in two dimensions, useful in tracking problems using microscopy (Dickinson and Tranquillo 1993) but with possible applications to for example satellite data (Farine et al. 2015). The key limitation here is that we consider the case of isotropic diffusion, i.e SDE:s where the diffusion matrix is given by $\sigma =\sqrt{2D}\textbf{I}$, where *D* is the diffusion coefficient. However, such a model is applicable in a diverse array of cases, e.g tracking of animal migration or bacterial movement (Browning et al. 2020).

The work in this paper pertaining to analytical expressions of approximate transition densities is in itself not new; there exists a wide literature on the subject that cover many different levels of approximation. See for example (Gobet and Pagliarani 2018) for a comprehensive treatise. What is lacking in the literature, however, is simple methods for deriving and using such transitions when facing realistic scientific problems, and thus our contribution is to provide a bridge between the field of stochastic calculus and mathematical biology.

## Setting and assumptions

In this section, we will specify what type of SDE models our method applies to. Consider a system of *N* interacting particles in $\textbf{R}^2$, with the system first being observed at time $t_k$. Individually, each particle $\textbf{x}_i(t)$:s time evolution is modelled as an autonomous SDE with isotropic diffusion; i.e

2.1 $$\begin{aligned} \textrm{d}\textbf{x}_i(t)&= \textbf{a}_i(\textbf{x}(t))\textrm{d}t + \Sigma _i\textrm{d}W_i(t), \end{aligned}$$

2.2 $$\begin{aligned} \textbf{x}_{i}(t_k)&= \textbf{x}_{ik}. \end{aligned}$$

where $t>t_k$, $\textbf{x}_i(t)\in \textbf{R}^2$, $\textbf{x}(t) = [\textbf{x}_1^T(t),\textbf{x}_2^T(t),\dots ,\textbf{x}_N^T(t)]^T$, $\Sigma _i=\sigma _i\textbf{I}$ is a $2\times 2$ diffusion matrix, $W_i(t)$ is a two-dimensional Wiener process and $\textbf{a}_i(\textbf{x}(t)):\textbf{R}^{2N}\mapsto \textbf{R}^2$ is a twice differentiable vector-valued function modelling the interaction of the particles. We assume that all interactions featured in $\textbf{a}_i$ are pairwise and uniform across all pairs of particles, i.e

2.3 $$\begin{aligned} \textbf{a}_i(\textbf{x}(t)) = \sum _{j\ne i}\textbf{a}(\Vert \textbf{x}_i(t)-\textbf{x}_j(t)\Vert ). \end{aligned}$$

Assume we observe the state of the particle system at equally spaced times $t_k$, $k=0,1,\dots ,K$ and from this, we wish to conduct inference on $\sigma _i$. For the context of this paper, we assume that $\textbf{a}$ is a known function.

## Method

### Brief overview of MSD

The typical way to compute the mean square displacement for a group of random walkers $\textbf{x}_i(t)$, $i=1,\dots ,N$ observed at the times $t_0$ and $T>t_0$ is by computing

3.1 $$\begin{aligned} \text {MSD}_{\textbf{x}}(T) = \frac{1}{N}\sum _{i=1}^N\Vert \textbf{x}_i(T)-\textbf{x}_i(t_0)\Vert ^2 \end{aligned}$$

If $\textbf{x}$ is the position of random walkers in *d* spatial dimensions, this quantity relates to the diffusion coefficient *D* for $\textbf{x}$ through

$$\begin{aligned} \text {MSD}_{\textbf{x}}(T) = 2dDT. \end{aligned}$$

Framing this in the context of SDE:s, if the random walkers $\textbf{x}_i(t)$ are independent and follows a pure Wiener process; i.e (1.1) with $\mu =0$ and $\sigma :=\sqrt{2D}$, we see that the expression (3.1) is simply the sample variance of the transition densities (Klebaner 2012)

3.2 $$\begin{aligned} \textbf{x}_{i}(T) \sim \mathcal {N}(\textbf{x}_i(t_0),TD\textbf{I}_{d\times d}) \end{aligned}$$

when sampled once for each random walker. Now, from independence of increments in Brownian motion, we can expand this notion given a set of observations $[\textbf{x}_{i0},\textbf{x}_{i1},\dots ,\textbf{x}_{iK}]$ with $\textbf{x}_{ik}:=\textbf{x}_i(t_{k})$ and uniform observation frequency $t_{k+1}-t_k:=\Delta t$ is by computing

3.3 $$\begin{aligned} \text {MSD}_{\textbf{x}}^*(T) = \frac{1}{N}\sum _{i=1}^N\sum _{k=0}^{K-1}\frac{\Vert \textbf{x}_{i(k+1)}-\textbf{x}_{ik}\Vert ^2}{\Delta t}. \end{aligned}$$

Here, $t_0 = 0$ and $t_K=T$. Crucially, one can note that the quantity MSD$^*_\textbf{x}(T)/d$ is in the fact the maximum likelihood estimator for *D*. This follows from the Gaussian increments of Wiener processes;

3.4 $$\begin{aligned} \textbf{x}_{i(k+1)} \sim \mathcal {N}(\textbf{x}_{ik},\Delta tD\textbf{I}_{d\times d}). \end{aligned}$$

We conclude by noting that given (3.4), we can recover the distribution (3.2) by filtering the sum of our observations with respect to only the first observation;

$$\begin{aligned}{} & {} \textbf{x}_i(T)|\textbf{x}_{i0}\sim \mathcal {N}(\textbf{x}_{i(K-1)},\Delta tD\textbf{I}_{d\times d})|\textbf{x}_{i0} \sim \mathcal {N}(x_{i(K-2)},\\{} & {} \quad 2\Delta tD\textbf{I}_{d\times d})|\textbf{x}_{i0} \sim \dots \sim \mathcal {N}(\textbf{x}_{i0},TD\textbf{I}_{d\times d}). \end{aligned}$$

This comes from the martingale property of Wiener processes (Klebaner 2012). *X*|*Y* should be read as "distribution of *X* given *Y* is known".

### Derivation of our method

The calculations carried out in this section are inspired by the framework given in Chapter 5 and 10 in Kloeden and Platen (1992), where the interested reader can find information on how to derive similar or higher order schemes for simulating SDE:s. The book does not go into details with applications to interacting particle systems, however, wherein our main contribution lies. For motivation, we will start by defining the basic Euler-Maruyama approximation of $$\textbf{x}_i(t)$$ on a time interval $$[t_k,t_{k+1})$$.

#### Definition 3.1

*(Euler-Maruyama with remainder term)*. Let $0\le t_k<t_{k+1}$ be a time interval and let $\textbf{x}_i(t)$ be a solution to the SDE (2.1). Let the particle state $\textbf{x}_i(t_k):=\textbf{x}_{ik}$ be known for all particles $i=1,\dots ,N$ and let $\textbf{a}_{ik}=\textbf{a}_i(\textbf{x}(t_k))$. The Euler Maruyama approximation $\hat{\textbf{x}}_i(t)$ of $\textbf{x}_i(t)$ with remainder $\mathcal {R}_1$ on this interval is given by

3.5 $$\begin{aligned} \hat{\textbf{x}}_i(t)&= \textbf{x}_{ik} + \textbf{a}_{ik}(t-t_k)+\sigma _i\int _{t_k}^t\textrm{d}W_i(s), \end{aligned}$$

3.6 $$\begin{aligned} \textbf{x}(t)-\hat{\textbf{x}}(t)&\sim \mathcal {R}_1=\int _{t_k}^t\Big [\int _{t_k}^s L_0\textbf{a}_i(\textbf{x}(z))\textrm{d}z + \int _{t_k}^s L_1\textbf{a}_i(\textbf{x}(z))\textrm{d}W_z \Big ]\textrm{d}s, \end{aligned}$$

3.7 $$\begin{aligned} L_0\textbf{a}_i(\textbf{x}(t))&= \textbf{a}_i(\textbf{x}(t))\nabla _{\textbf{x}_i}\textbf{a}_i(\textbf{x}(t))+\frac{\sigma _i^2}{2}\nabla _{\textbf{x}_i}^2\textbf{a}_i(\textbf{x}(t)) \end{aligned}$$

3.8 $$\begin{aligned} L_1\textbf{a}_i(\textbf{x}(t))&= \sigma _i\nabla _{\textbf{x}_i}\textbf{a}_i(\textbf{x}(t)). \end{aligned}$$

Gradients are to be interpreted as Jacobian matrices;

$$\begin{aligned} \nabla _{\textbf{x}_i}\textbf{a}_i(\textbf{x}(t))=\begin{bmatrix}\partial _{\textbf{x}_{i1}}\textbf{a}_{ik1}(\textbf{x}(t)) &{} \partial _{\textbf{x}_{i2}}\textbf{a}_{ik1}(\textbf{x}(t)) \\ \partial _{\textbf{x}_{i1}}\textbf{a}_{ik2}(\textbf{x}(t)) &{} \partial _{\textbf{x}_{i2}}\textbf{a}_{ik2}(\textbf{x}(t))\end{bmatrix}:=\textbf{A}_i(t). \end{aligned}$$

Note that (3.5) can be stated as

$$\begin{aligned} \hat{\textbf{x}_i}(t) \sim \mathcal {N}(\textbf{x}_{ik}+\textbf{a}_{ik}(t-t_k),(t-t_k)\sigma _i^2 \textbf{I}_{2\times 2}). \end{aligned}$$

We use this as a stepping stone to the higher order approximation used in this paper, that will be presented in the following theorem.

#### Theorem 3.1

(Higher-order approximation for isotropic diffusion). Consider the system described by equation (2.1). Let the particle state $$\textbf{x}_i(t_k):=\textbf{x}_{ik}$$ be known for all particles $$i=1,\dots ,N$$, and let $$\textbf{A}_{ik}=\textbf{A}_i(t_k)$$. On the interval $$[t_k,t_{k+1})$$, we have a strong (pathwise) approximation $$\tilde{\textbf{x}}_i(t)$$ of $$\textbf{x}_i(t)$$ given by

3.9 $$\begin{aligned} \tilde{\textbf{x}}_i(t)&\sim \mathcal {N}(\textbf{m}_{ik}(t),\textbf{S}_{ik}(t)),\nonumber \\ \textbf{m}_{ik}(t)&= \textbf{x}_{ik} + \textbf{a}_{ik}(t-t_k),\nonumber \\ \textbf{S}_{ik}(t)&= \textbf{S}_{1ik}^T(t)\textbf{S}_{1ik}(t)+\textbf{S}_{2ik}^T(t)\textbf{S}_{2ik}(t),\nonumber \\ \textbf{S}_{1ik}(t)&= \sigma _i\sqrt{t-t_k}(\textbf{I}+\frac{t-t_k}{2}\textbf{A}_{ik}),\qquad \textbf{S}_{2ik}(t) = \sigma _i\frac{(t-t_k)^{\frac{3}{2}}}{\sqrt{12}}\textbf{A}_{ik}. \end{aligned}$$

#### Proof

The trick is to use Itô’s lemma on $\textbf{A}_i(t)=\nabla _{\textbf{x}_i}\textbf{a}_i(\textbf{x}(t))$, and apply this to its occurrence in the $L_1$ featured in (3.6);

3.10 $$\begin{aligned} \textbf{A}_i(t) = \underbrace{\textbf{A}_i(t_k)}_{\textbf{A}_{ik}} + \int _{t_k}^tL_0\textbf{A}_i(s)\textrm{d}s +\int _{t_k}^t L_1\textbf{A}_i(s)\textrm{d}W_i(s) \end{aligned}$$

We then plug (3.10) into the $\mathcal {R}_1$ in (3.6) and get that

3.11 $$\begin{aligned} \textbf{x}_i(t)-\hat{\textbf{x}}(t) \sim \quad&\sigma _i\textbf{A}_{ik}\int _{t_k}^t W_i(s)\textrm{d}s\nonumber \\&+ \int _{t_k}^t\Big [\int _{t_k}^s L_0\textbf{a}_i(\textbf{x}(z))\textrm{d}z + \int _{t_k}^s \Big [\int _{t_k}^z L_1L_0\textbf{a}_i(\textbf{x}(u)) \textrm{d}u \end{aligned}$$

3.12 $$\begin{aligned}&+\int _{t_k}^z L_1L_1\textbf{a}_i(\textbf{x}(u)) \textrm{d}W_u\Big ]\textrm{d}W_z\Big ]\textrm{d}s.\nonumber \\&\Longrightarrow \nonumber \\ \textbf{x}_i(t)&= \textbf{x}_{ik} + \textbf{a}_{ik}(t-t_k)+\sigma _i\int _{t_k}^t\textrm{d}W_i(s)+\sigma _i\textbf{A}_{ik}\int _{t_k}^t W_i(s)\textrm{d}s +\mathcal {R}_2 \end{aligned}$$

where $\mathcal {R}_2$ is a remainder term consisting of (3.11)–(3.12). This gives rise to the the higher order scheme

3.13 $$\begin{aligned} \tilde{\textbf{x}}_i(t) = \textbf{x}_{ik} + \textbf{a}_{ik}(t-t_k)+\sigma _i\underbrace{\int _{t_k}^t\textrm{d}W_i(s)}_{:=Z_1(t)}+\sigma _i\textbf{A}_{ik}\underbrace{\int _{t_k}^t W_i(s)\textrm{d}s}_{:= Z_2(t)} \end{aligned}$$

We now see that there are two sources of randomness when propagating from the state $\textbf{x}_{ik}$ to $\textbf{x}_i(t)$ using the approximation (3.13), $Z_1(t)$ and its integral process $Z_2(t)$. We use the fact that $Z_1(t)$ has the known distribution $Z_1(t)\sim \mathcal {N}(\textbf{0},(t-t_{k})\textbf{I})$ to deduce the distribution of $Z_2(t)$ and its relationship to $Z_1(t)$. Note first in $Z_1(t)$ that it has no correlation structure in its two spatial dimensions, and thus we can carry out the upcoming calculations in parallel for the marginal distributions. We will use the well-established trick to apply Itô’s lemma in one dimension on the function *tW*(*t*);

$$\begin{aligned} \textrm{d}(tW(t))&= W(t)\textrm{d}t+t\textrm{d}W(t) \Rightarrow \\ t\int _{t_k}^t\textrm{d}W(s)&= \int _{t_k}^tW(s)\textrm{d}s+\int _{t_k}^ts\textrm{d}W(s) \Rightarrow \\ \int _{t_k}^{t}(t-s)\textrm{d}W(s)&= \int _{t_k}^tW(s)\textrm{d}s ; \end{aligned}$$

to arrive at the conclusion that $Z_2(t)\sim \mathcal {N}(\textbf{0},\frac{1}{3}(t-t_{k})^3\textbf{I})$ by using Itô isometry to find the variance of the process $\int _{t_k}^t(t-s)\textrm{d}W(s)$. Next, we find the covariance of $Z_1(t)$ and $Z_2(t)$, once again by Itô isometry;

$$\begin{aligned} \textbf{E}[Z_1(t)Z_2(t)]&=\textbf{E}\Big [\int _{t_k}^t\textrm{d}W(s)\int _{t_k}^tW(s)\textrm{d}s\Big ]\\&= \textbf{E}\Big [\int _{t_k}^t\textrm{d}W(s)\int _{t_k}^t(t-s)\textrm{d}W(s)\Big ]=\int _{t_k}^t(t-s)\textrm{d}s = \frac{1}{2}(t-t_k)^2. \end{aligned}$$

Thus, we arrive at the conclusion that $Z_1(t)$ and $Z_2(t)$ can be expressed as a linear combination of two independent standard normal random variables $U_1$ and $U_2$ given a time $t\in [t_k,t_{k+1})$;

3.14 $$\begin{aligned} Z_1(t)&= \sqrt{t-t_k}U_1 \end{aligned}$$

3.15 $$\begin{aligned} Z_2(t)&= \frac{(t-t_k)^{\frac{3}{2}}}{2}\big (U_1+\frac{1}{\sqrt{3}}U_2\big ). \end{aligned}$$

By substituting $Z_{1}$ and $Z_2$ in (3.13) with (3.14)–(3.15), we find that $\textbf{x}_i(t)$ follows a Gaussian distribution $\mathcal {N}(\textbf{m}_{ik}(t),\textbf{S}_{ik}(t))$, with

$$\begin{aligned} \textbf{m}_{ik}(t)&= \textbf{x}_{ik} + \textbf{a}_{ik}(t-t_k),\\ \textbf{S}_{ik}(t)&= \textbf{S}_{1ik}^T(t)\textbf{S}_{1ik}(t)+\textbf{S}_{2ik}^T(t)\textbf{S}_{2ik}(t),\\ \textbf{S}_{1ik}(t)&= \sigma _i\sqrt{t-t_k}(\textbf{I}+\frac{t-t_k}{2}\textbf{A}_{ik}),\qquad \textbf{S}_{2ik}(t) = \sigma _i\frac{(t-t_k)^{\frac{3}{2}}}{\sqrt{12}}\textbf{A}_{ik}. \end{aligned}$$

$$\square $$

#### Lemma 3.2

(Non-degeneracy of the estimate). For symmetric matrices $\textbf{A}_{ik}$, (3.9) constitutes a proper probability distribution.

#### Proof

We will prove this by showing that symmetric matrices $\textbf{A}_{ik}$ indicate a symmetric and positively definite matrix $\textbf{S}_{ik}(t)$, thus satisfying the requirement for $\textbf{S}_{ik(t)}$ to be a covariance matrix. We do this by showing that the smallest eigenvalue $\lambda _m$ of $\textbf{S}_{ik(t)}$ is positive. Set $\Delta t = t-t_k$, $a_{ij}$ as the (*i*, *j*):th element of $\textbf{A}_{ik}$ and shorthand $\textbf{S}_{ik}(t):=\textbf{S}_{ik}$. From a lengthy but conceptually simple calculation, we arrive at the following statements;

3.16 $$\begin{aligned} \lambda _m&= \frac{\text {Tr}(\textbf{S}_{ik})-\sqrt{\text {Tr}(\textbf{S}_{ik})^2-4|\textbf{S}_{ik}|}}{2},\nonumber \\ \text {Tr}(\textbf{S}_{ik})&= \frac{\sigma _i^2 \Delta t}{3}\Big [\frac{3}{2}+\big (\frac{3}{2}+\Delta ta_{11}\big )^2 + \big (\frac{3}{2}+\Delta ta_{22}\big )^2 + 2(\Delta t a_{12})^2 \Big ],\nonumber \\ |\textbf{S}_{ik}|&= \frac{\sigma _i^4(\Delta t)^2}{9}\Big [\Big (\frac{3}{4} +\big (\frac{3}{2}+\Delta ta_{11}\big )^2 +(\Delta t a_{12})^2\Big )\Big (\frac{3}{4} +\big (\frac{3}{2}+\Delta ta_{22}\big )^2 +(\Delta t a_{12})^2\Big )\nonumber \\&\quad -\Big (\big (3+\Delta ta_{11}+\Delta ta_{22}\big )\Delta t a_{12}\Big )^2\Big ] \Longrightarrow \nonumber \\ \text {Tr}(\textbf{S}_{ik})^2-4|\textbf{S}_{ik}|&=\big ((\Delta t a_{11})^2-(\Delta t a_{22})^2+3(a_{11}-a_{22})\big )^2\nonumber \\&\quad +4(\Delta t a_{12})^2\big (3+\Delta ta_{11}+\Delta ta_{22}\big )^2\ge 0. \end{aligned}$$

Since $\text {Tr}(\textbf{S}_{ik})^2-4|\textbf{S}_{ik}|\ge 0$, we will have positive $\lambda _m$ if and only if $|\textbf{S}_{ik}| >0$, i.e if

$$\begin{aligned}{} & {} \Big (\frac{3}{4} +\big (\frac{3}{2}+\Delta ta_{11}\big )^2 +(\Delta t a_{12})^2\Big )\Big (\frac{3}{4} +\big (\frac{3}{2}+\Delta ta_{22}\big )^2 +(\Delta t a_{12})^2\Big )>\\{} & {} \quad \Big (\big (3+\Delta ta_{11}+\Delta ta_{22}\big )\Delta t a_{12}\Big )^2 \end{aligned}$$

Set $\mu =\frac{3}{2}+\Delta ta_{11}$, $\nu =\frac{3}{2}+\Delta ta_{22}$ and $\omega = \Delta t a_{12}$ as a shorthand notation. The condition $|\textbf{S}_{ik}|>0$ can then be written as

$$\begin{aligned}&\big (\frac{3}{4}+\mu ^2+\omega \big )\big (\frac{3}{4}+\nu ^2+\omega \big )>\big ((\mu +\nu )\omega \big )^2\\&\qquad \qquad \qquad \qquad \qquad \Longleftrightarrow \\&12(\mu ^2+\nu ^2)+16(\omega ^2+\frac{3}{4}-\mu \nu )^2+9>0 \end{aligned}$$

which is trivially true, as only squares appear on the left hand side. $\square$

Now let us define $\overline{\textbf{S}_{ik}}=\sigma _i^{-2}\textbf{S}_{ik}(t_{k+1})$. We will use this to construct a likelihood function for the $k+1$:th observation of particle *i* using the *k*:th observation of all particles.

#### Corollary 3.2.1

(A conjugacy for isotropic diffusion). Assume we have *K* observations of *N* particles. Denote by $\tau _i:=\sigma ^{-2}_i$ the precision coefficient of the *i*:th particle. By imposing a prior distribution $\tau _i\sim \text {Gamma}(\alpha _0,\beta _0)$, we find the conjugate relationship

$$\begin{aligned} \alpha _K&= \alpha _0 + K,\\ \beta _K&= \beta _0 + \frac{1}{2}\sum _{k=0}^{K-1}\big (\textbf{x}_{i(k+1)}-\textbf{m}_{ik}\big )^T\overline{\textbf{S}}_{ik}^{-1}\big (\textbf{x}_{i(k+1)}-\textbf{m}_{ik}\big ) \end{aligned}$$

for the posterior distribution $p(\tau _i|\textbf{x}_{(1:K)},\theta )$.

#### Proof

The proof of this corollary is a straight-forward computation using the result of Theorem 3.1. From one observation *k* to the next, we have the following likelihood function;

3.17 $$\begin{aligned} p(\textbf{x}_{i(k+1)}|\textbf{x}_k) = (2\pi \sigma _i\sqrt{|\overline{\textbf{S}_{ik}}|})^{-1} \exp {\big (-\frac{1}{2\sigma _i^2}\big (\textbf{x}_{i(k+1)}-\textbf{m}_{ik}\big )^T\overline{\textbf{S}_{ik}}^{-1}\big (\textbf{x}_{i(k+1)}-\textbf{m}_{ik}\big )\big )}.\nonumber \\ \end{aligned}$$

Taking the logarithm of (3.17) and summing over all *K* observations gives us the log-likelihood for the entire sequence of observations;

3.18 $$\begin{aligned} \ell {(\textbf{x}_{i(1:K)})}\propto \frac{K}{2}\log {(\sigma _i^{-2})}-\sigma _i^{-2}\frac{1}{2}\sum _{k=0}^{K-1}\big (\textbf{x}_{i(k+1)}-\textbf{m}_{ik}\big )^T\overline{\textbf{S}_{ik}}^{-1}\big (\textbf{x}_{i(k+1)}-\textbf{m}_{ik}\big ).\qquad \end{aligned}$$

We now see that with $\sigma _i^{-2}:=\tau _i$, this is the log likelihood for

$$\begin{aligned} \tau _i\sim \text {Gamma}\big (1+K,\frac{1}{2}\sum _{k=0}^{K-1}\big (\textbf{x}_{i(k+1)}-\textbf{m}_{ik}\big )^T\overline{\textbf{S}_{ik}}^{-1}\big (\textbf{x}_{i(k+1)}-\textbf{m}_{ik}\big )\big ) \end{aligned}$$

giving us the result stated in the corollary. $\square$

For frequentist statistics, we can instead use the maximum likelihood estimate for $\sigma _i$,

3.19 $$\begin{aligned} \hat{\sigma }_i^2 = \frac{\sum _{k=0}^{K-1}\big (\textbf{x}_{i(k+1)}-\textbf{m}_{ik}\big )^T\overline{\textbf{S}_{ik}}^{-1}\big (\textbf{x}_{i(k+1)}-\textbf{m}_{ik}\big )}{2K}. \end{aligned}$$

Note now that in the case of $\textbf{a}= \textbf{0}$, (3.19) reduces to the MSD maximum likelihood as stated in (3.3). Another question worth discussing is whether or not further improvements can be made and still keep the conjugacy properties that the introduced method enjoys. For this, we need to take a closer look at the remainder term $\mathcal {R}_2$ introduced in (3.11)–(3.12). Explicitly written, we have that

$$\begin{aligned} \mathcal {R}_2(t) = \int _{t_k}^t\Big [\int _{t_k}^s L_0\textbf{a}_i(\textbf{x}(z))\textrm{d}z + \int _{t_k}^s \Big [\int _{t_k}^z L_1L_0\textbf{a}_i(\textbf{x}(u)) \textrm{d}u \int _{t_k}^z L_1L_1\textbf{a}_i(\textbf{x}(u)) \textrm{d}W_u\Big ]\textrm{d}W_z\Big ]\textrm{d}s. \end{aligned}$$

From (3.7)–(3.8), we have that $\sigma _i$ appears in powers of at least two in $\mathcal {R}_2$, and for the triple integral $\int _{t_k}^t\int _{t_k}^s\int _{t_k}^z L_1L_0\textbf{a}_i(\textbf{x}(u))\textrm{d}u\textrm{d}W_z \textrm{d}s$, $\sigma _i$ appears in a power of three. Since the conjugacy is founded on linear appearances of $\sigma _i$, we have that the conjugacy covered in this paper is the most exact conjugate relationship available for isotropic diffusion coefficients in interacting systems of stochastic differential equations. Conditions on when the higher order scheme improves upon the Euler-Maruyama scheme can be found in the supplementary material.

## Application to in vitro cancer cell migration

**Fig. 2:**
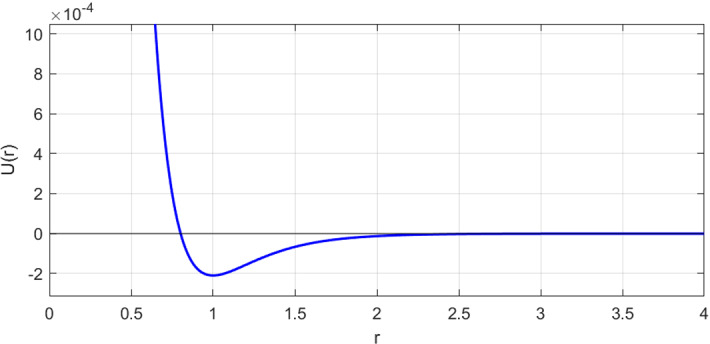
Typical profile of our adhesion-repulsion potential (4.3), here with parameters $D_e = 0.00021$, $a=3.5$ and $\varphi (r)=e^{-r}$

**Fig. 3:**
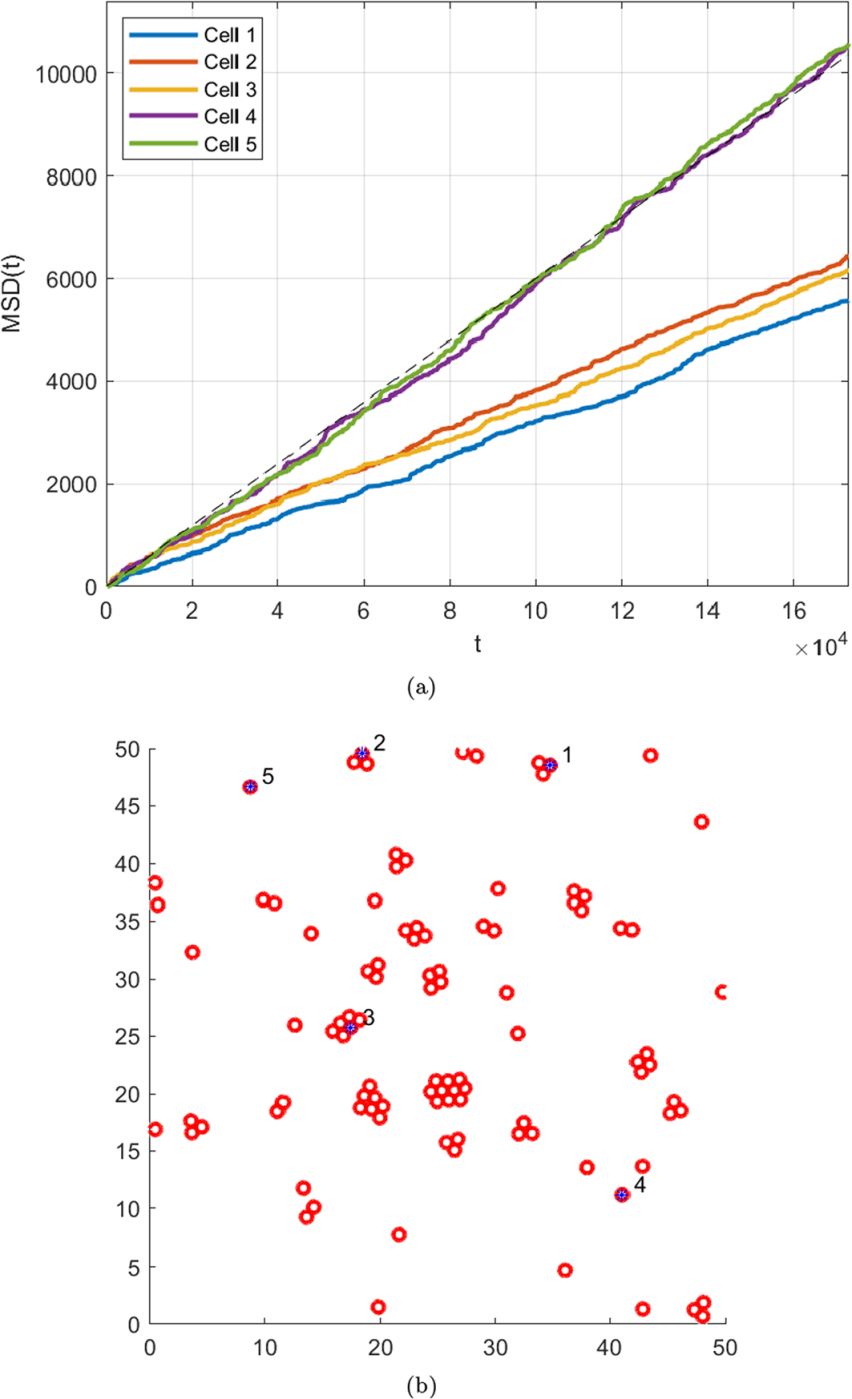
An example of how repulsive-attractive particle systems induce sub-diffusive behaviour on particles with more neighbours. Here, every single particle of the 100 generated have the same inate diffusion coefficient, and the MSD of the two particles free from neighbours reflect the “true” diffusivity. The parameters used to generate this dataset is given as Experiment 0 in Table 1

We now return to our main interest, which is applying this method to models for in vitro cell migration. We chose to model our cell population using a system of interacting stochastic differential equations with isotropic diffusion, where the interactions are *attractive-repulsive*. It has however been observed that some cells are more motile than others (Kwon et al. 2019), and as such we choose to give every cell indexed by *i* its own diffusion coefficient $\sigma _i$. At a particular moment in time *t*, the system evolves according to the following set of equations

4.1 $$\begin{aligned} \textrm{d}\textbf{x}_i&= -\nabla V(\textbf{x}_i,t)\textrm{d}t + \Sigma _i\textrm{d}W_i(t), \end{aligned}$$

4.2 $$\begin{aligned} V(\textbf{x},t)&=\sum _{j=1}^{N_t} U(\Vert \textbf{x}-\textbf{x}_j\Vert ), \end{aligned}$$

4.3 $$\begin{aligned} U(r)&= D_e\Big [1 -\Big (\frac{\varphi (r)}{\varphi (r_0)}\Big )^a\Big ]^2-D_e. \end{aligned}$$

where $\varphi (r):\textbf{R}^+\mapsto \textbf{R}^+$ is a positive, monotonically decreasing function so that $\lim _{r\rightarrow \infty }\varphi (r)=0$. We chose $\varphi (r)=e^{-r}$ - this choice of $\varphi$ gives us the *Morse potential* as our model of interactions; an example of of this potential is visualized in Fig. 2. Other choices such as $\varphi (r)=1/r$ are viable as well, and that particular $\varphi$ gives us the Lennard–Jones potential. $r_0$ is the equilibrium distance between two cells. Since the setting of this study is on a microscopic length scale, we fix $r_0=1$. That makes it so that the entire interaction potential is governed by just $D_e$ (well depth) and *a* (well steepness). In the language of the general case covered in Sect. 3, we have the following vectors and matrices;

$$\begin{aligned} \textbf{a}_{ik}&= -\nabla V(\textbf{x}_i,t),\qquad \Sigma _i = \sigma _i \textbf{I},\\ \textbf{A}_{ik}&= -\sum _{j\ne i}\text {Hessian}(U(\textbf{x}_{ik}-\textbf{x}_{jk})). \end{aligned}$$

### Numerical experiment

To display the improvements in inference on the diffusion coefficient a number of *in silico* experiments were performed. The experiments are designed to mimic the behavior of *glioblastoma multiforme* cancer cells migrating in vitro. Particle systems were generated from the model (4.1). We use a simulation time step $h=1$ corresponding to one second. Cell migration is a slow process, and a typical diffusion coefficient in the setting we are simulating is 0.0013–0.0065 [cm$^2$/day] (Swanson et al. 2000). Thus, $h = 1$ second is “close to continuous” given the scale of the problem. For the sake of simplicity, we express the diffusion coefficient in the unit [average cell diameter$^2$/second], since the length scale of the simulation is set so that [average cell diameter] $=1$. In Fig. 3, we see a snapshot from a data set generated using the model on the right. On the left, we see the evolution of the MSD over the entire time span for five tagged cells. All of these cells were seeded with the same diffusion coefficient, but they display widely varying MSD outcomes, stemming from interactions with neighbouring cells.

Four distinct experiments were performed to illustrate how our method improves on using MSD to estimate diffusivity in interacting particle systems. The experiments were designed to capture two dimensions of interest, namely the effects of temporal resolution and the effects of cell density on the accuracy of diffusion estimation accuracy. Experiment *n*, for $n=1,\dots 4$, examines a particle system of an increasing number of individuals seeded uniformly in a $40\times 40$ square, and confined by hard boundaries in a $50\times 50$ square area. The interaction parameters and diffusion coefficients are the same for all these experiments, see again Table 1. We then observe this particle system every 5, 10, 15, 20, 30, 45 and 60 min over the course of two days. Pseudo-code for implementation of the inference algorithm is available in the supplementary material, as well as a GitHub repository containing all code needed to reproduce the figures.

**Table 1:** Parameters used for simulation

	$D_e$	*a*	$\sigma$	*N*
Experiment 0	0.0004	4	$10^{-2}$	100
Experiment *n*	0.00021	3.5	$e^{-9/2}$	$2^{5+n}$

### Results

**Fig. 4:**
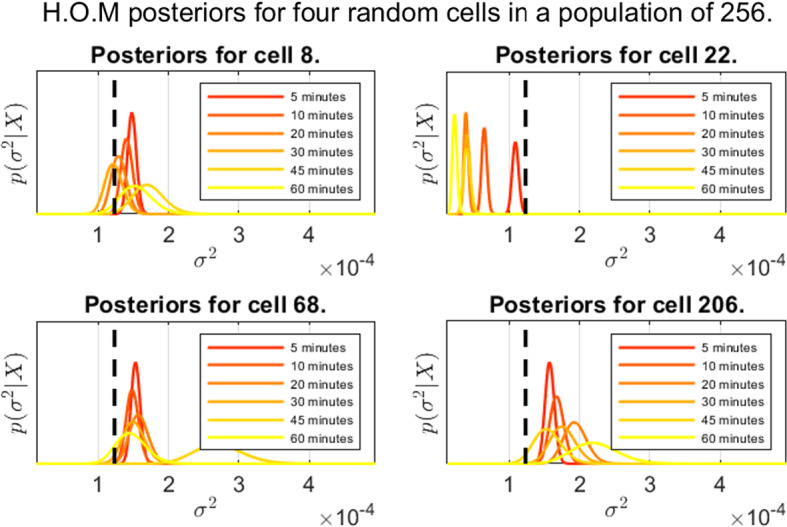
Representative posterior distributions using the higher order method as given by Corollary 3.2.1 with prior $\alpha _0=\beta _0=0$. Ground truth in black

In this section, we present the results from the experiments detailed in Table 1. Throughout all experiments, an improper prior of $\alpha _0=\beta _0=0$ is used. In Fig. 4, we see the posterior distributions for four randomly selected cells from the experiment featuring 256 cells (experiment 3), along with a black dashed line marking the ground truth. Here, we note some heteroscedacity in the estimates, both across the population and how observation frequency plays in. In general, we see a pattern of higher variability in accuracy (i.e mode deviation from the true value, marked with a black dashed line) as the inter-observation time increases, with the expected increase in posterior variance (due to fewer samples) also playing a role.

**Fig. 5:**
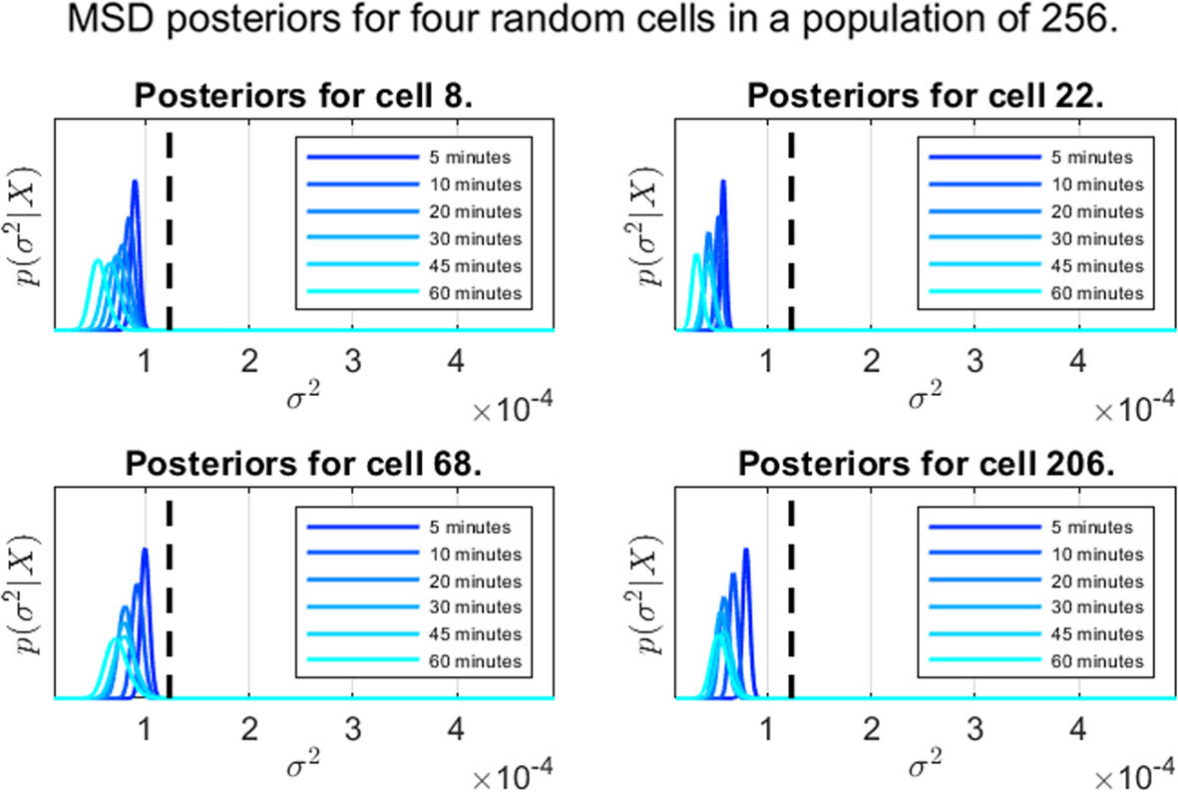
Representative posteriors corresponding to MSD, generated using the assumption (3.4) instead of the higher order estimate given by Theorem 3.1. Prior parameters $\alpha _0=\beta _0=0$. Ground truth in black

Figure 5 shows the ‘MSD posterior’ for all cells; i.e a Bayesian approach where assume that the observation $\textbf{x}_{i(k+1)}$ given $\textbf{x}_{ik}$ follow the distribution (3.4), giving us a Gamma posterior. Here, the variability in both posterior precision and posterior variance are significantly lower. However, there is a systematic error in the posterior distributions, in line with the observations made in Fig. 3. The diffusion coefficents are predictably underestimated, unlike the case in Fig. 4 where much higher posterior precision is observed, at least for frequent observations.

**Fig. 6:**
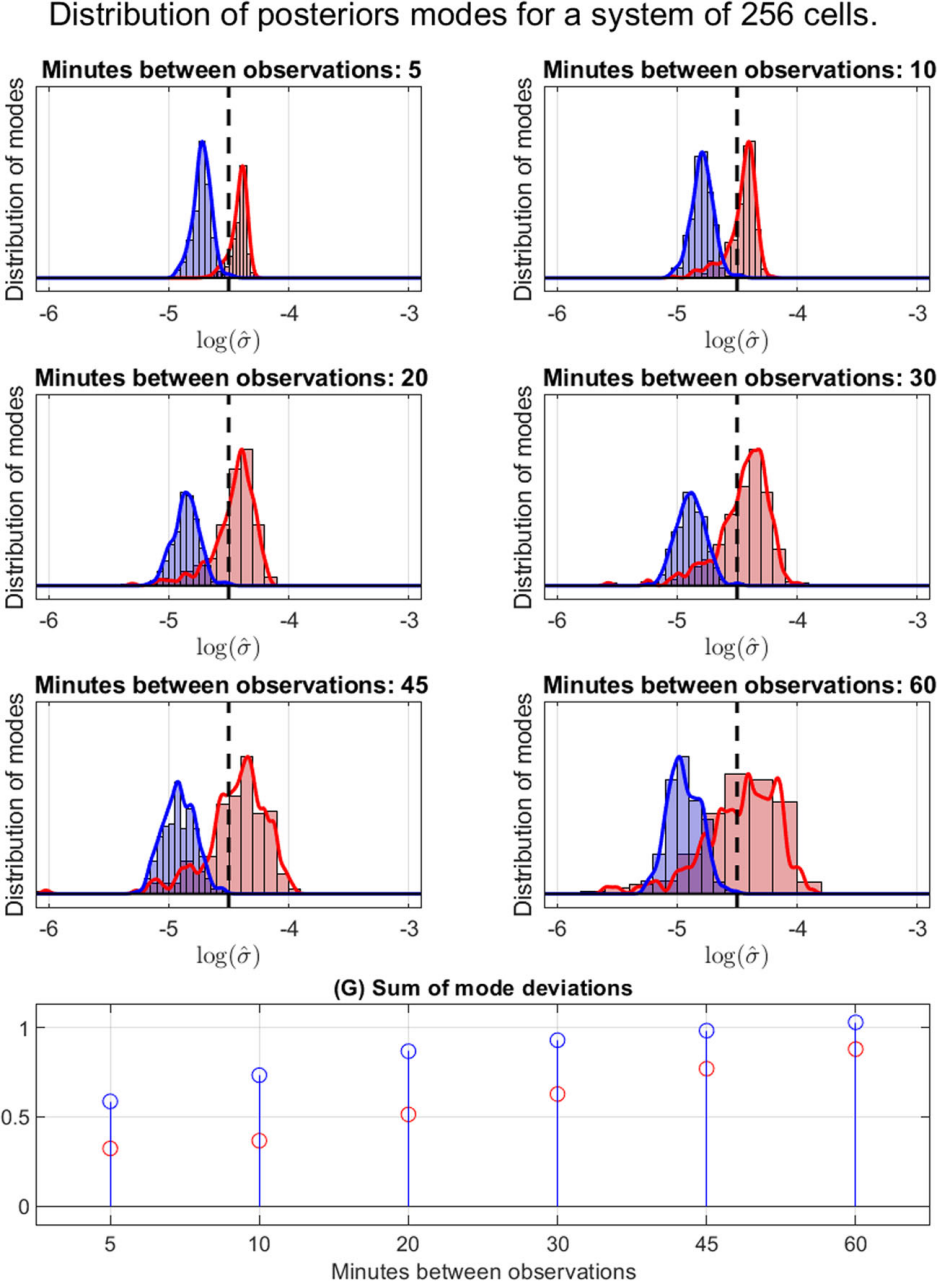
Detailed statistics for the results of experiment 3. Although the variance in mode accuracy compared to the ground truth (black) increases for our method (shown in red) for infrequent observations, the sum of mode deviations is consistently lower than for using MSD (displayed in blue). The kernels used for smoothing are normal with standard deviation $N^{-5/8}$, in accordance with optimal bandwidth theory (Chen 2017)

**Fig. 7:**
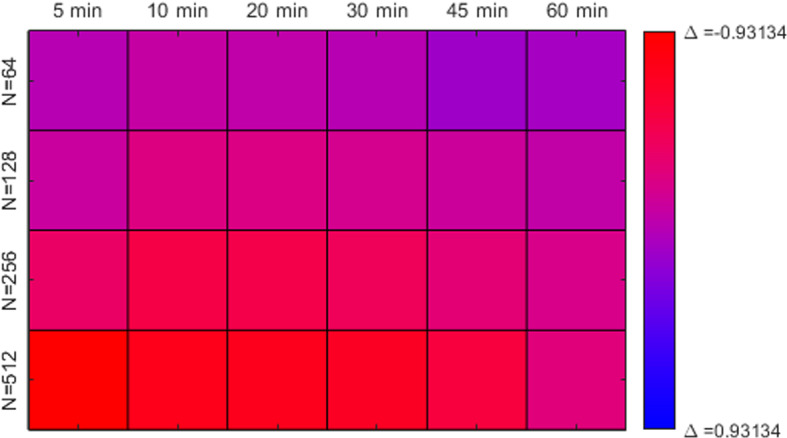
Summary comparison of our model to using MSD for experiment 1–4 at all observation frequencies. More red shades correspond to better performance when using our method, more blue shades better performance for MSD

To explore the performance of our method as compared to using MSD, we consider the distribution of the *modes* of the posteriors, as shown in Fig. 6. In the first six panels, we show kernel smoothed histograms of the $\log$ modes $\hat{\sigma }$ of the posterior distributions for *every* cell in experiment 3 for different time resolutions. We display the results from estimating the diffusion coefficient using MSD in blue and with our method in red. Here, the global trend hinted at through Figs. 4 and 5 is in full display; we see a systematic error in estimating $\sigma$ using MSD, with much better accuracy (although at times more variance) using our method. In panel (G), we present our measurement of model performance, the *sum of mode deviations*. Remembering that we have a ground truth of $\sigma = e^{-9/2}$, we display the sums

4.4 $$\begin{aligned} E_{\text {MSD}}&= \Big (\sum _{i=1}^N(\sigma - \hat{\sigma }_{i\text {MSD}} )^2\Big )^{1/2}, \end{aligned}$$

4.5 $$\begin{aligned} E_{\text {HOM}}&= \Big (\sum _{i=1}^N(\sigma - \hat{\sigma }_{i\text {HOM}} )^2\Big )^{1/2} \end{aligned}$$

in blue and red respectively, where $E_{\text {MSD}}$ is the error for using MSD and $E_{\text {HOS}}$ is the error for using the higher order scheme (our method). A consistently better performance for the higher order scheme can be seen across all temporal reoslutions considered.

We finish the presentation of model performances compared to one another by considering the quantity

$$\begin{aligned} \Delta = E_{\text {MSD}} - E_{\text {HOM}} \end{aligned}$$

for each of our experiments at all temporal resolutions, summarized in Fig. 7. Here, every square represents a cell density, given by the rows, and a temporal resolution, given by the columns. The shade of the square corresponds to $\Delta$. Large negative values of $\Delta$ corresponds to an advantage of our method comapred to MSD. For the particular datasets used to generate these figures, the (in absolute terms) largest difference was $\Delta = -0.93134$, and thus the coloring use this difference as a benchmark. The performance when using MSD was superior to our method for only two cases; the cases of 64 cells observed for 45 and 60 min respectively. Accordingly, these squares have the bluest shade of purple, and all other squares takes a shade of purple featuring more red hues. The dataset, along with all code, is available at the GitHub repositories https://github.com/GustavLW/Inference along with https://github.com/GustavLW/Simulation.

## Discussion

In this paper, we have proposed a solution to the problem of estimating diffusion coefficients in systems with strong inter-particle interactions that relies on the existence of a model of the inter-particle dynamics. We achieve this by expanding the standard Euler-Maruyama scheme to account for these particle interactions.

First we consider the computational complexity of the algorithm as seen in the supplementary material. Both Euler-Maruyama and the higher order method has a worst case complexity scaling of $\mathcal {O}(KN^2)$. Upon closer inspection, however, we see that there are about four times as many calculations going into the higher order method (counting all that goes into line 11–13 in Algorithm 1). On the other hand, we have observations of computational performance improvement that by far outweighs this drawback, especially so for particle systems of higher density (see Fig. 7). From the example provided in Fig. 6, we see that the sum of mode deviations for 30 min time resolution using our method is comparable to the 5 min intervals when using MSD.

The application of this process has great potential use in future studies of in vitro cell cultures in particular. It has been noted that in order for a cell culture to remain viable in a laboratory setting, a certain cell density need to be maintained (Gerlee et al. 2022). If one now wishes to estimate the diffusivity of the cells under such circumstances, our simulation study shows ample evidence that MSD is insufficient due to crowding effects. As such, correcting the diffusion estimation at the modest cost of using a model of the interaction is of great interest to mathematicians and biologists alike.

An unavoidable drawback of our method is the requirement of well defined derivatives of the interaction function $\textbf{a}$, meaning that we are still somewhat limited in what methods we can apply this method to. For example, in purely hard-sphere interactions, which is a popular model for ideal gases (Krapivsky et al. 2010), the first derivative of the interaction term is not well defined along the surface of the sphere. One can circumvent this by for example smoothing the interaction kernel, but the risk remains of numerical issues. Alas, this method is best served by models with soft and smooth interactions, as is common in biology and ecology (Lewis et al. 2013; Oelschläger 1989).

There is an intrinsic relationship between interacting diffusion and the phenomena of sub- and superdiffusion, a phenomena observed both theoretically and experimentally (Stauffer et al. 2007). Anomalous diffusion can emerge in a number of ways from the stand-point of stochastic calculus. On one hand, it can be a deliberate design choice of the model to choose a driving noise with covariance structure different from that of the Wiener process (Benhamou 2007). It can also be emergent from the interaction, e.g an emphasis on repulsive interactions will result in superdiffusive behaviour even when the underlying noise is Brownian (Fedotov and Korabel 2017). In the latter case, the 0.5-order approximation of Euler-Maruyama fails to take this phenomena into account by its very construction. This could be one potential reason why superdiffusion has been observed in crowded environment when naive MSD-methods have been utilized (Smith et al. 2017). The higher order method, however, adjusts the diffusivity by taking the Jacobian matrix of the interaction into account, making inference on the underlying, normal diffusion possible even in the case of seemingly anomalous diffusion.

The method presented in this paper can be combined with other inference strategies to conduct inference on large SDE systems. If other conjugacies exists in the drift term, one could for example construct a Gibbs sampler that alternates inference on the drift parameters and the diffusion coefficients. For less tractable models, one could still divide the inference into blocks, using conjugacies for the diffusion term and likelihood-free inference for other aspects of the model, such as the use of particle filters (Schön and Lindsten 2015). Methods such as these are proven to converge, but the mixing time of such Markov chain Monte Carlo (MCMC) methods are notoriously difficult to study, and convergence can thus be slow beyond feasibility.

It should be noted that the method presented in this paper still relies on a linearisation of the drift term. For frequent observations this is an reasonable approximation, but for infrequent observations this can lead to inaccuracies, as noted in Fig. 7 where our method performed worse than standard MSD for infrequent observations of sparse particle systems. A way to remedy this can be to, instead of assuming constant drift terms between the observations, solving an ODE for the expected value and the variance of each particle location on the interval between observations. While this leads to further computational complexity, it makes the method less sensitive to infrequent observations and is an avenue of further research. Such methods are discussed in detail in for example (Särkkä 2013).

To summarise, we have shown that more exact conjugacies exists given systems satisfying some fairly basic smoothness requirements. The application in mind when this discovery was made was interacting particle systems, but applications can be found in many other settings where accurate inference on a diffusion coefficient in a complex system is of importance, such as finance.

## Supplementary Information

Below is the link to the electronic supplementary material.

Supplementary file 1 (pdf 254 KB)
