# Supplementary material for: Fast and precise inference on diffusivity in interacting particle systems
Source: J Math Biol. 2023 Mar 29;86(5):64. doi: 10.1007/s00285-023-01902-y (PMC10060353; doi:10.1007/s00285-023-01902-y)
Supplement: Supplementary file 1 — (pdf 254 KB) [file 285_2023_1902_MOESM1_ESM.pdf]

Fast and precise inference on diffusivity in interactive particle systems - supplementary material

Gustav Lindwall[*], Philip Gerlee[†]

August 25, 2022

A Setting and assumptions

Consider a system of N interacting particles in $\mathbf{R}^2$, with the system first being observed at time t_k. Individually, each particle $\mathbf{x}_i(t)$:s time evolution is modelled as an autonomous SDE with isotropic diffusion; i.e

$$\mathrm{d}\mathbf{x}_i(t) = \mathbf{a}_i(\mathbf{x}(t))\mathrm{d}t + \Sigma_i \mathrm{d}W_t, \tag{A.1}$$
$$\mathbf{x}_i(t_k) = \mathbf{x}_{ik}. \tag{A.2}$$

where $t > t_k$, $\mathbf{x}_i(t) \in \mathbf{R}^2$, $\mathbf{x}(t) = [\mathbf{x}_1^T(t), \mathbf{x}_2^T(t), \ldots, \mathbf{x}_N^T(t)]^T$, $\Sigma_i = \sigma_i \mathbf{I}$ is a 2×2 diffusion matrix, $W_i(t)$ is a two-dimensional Wiener process and $\mathbf{a}_i(\mathbf{x}(t)) : \mathbf{R}^{2N} \mapsto \mathbf{R}^2$ is a twice differentiable vector-valued function modelling the interaction of the particles. More precisely, we assume that all interactions featured in $\mathbf{a}_i$ are pairwise and uniform across all pairs of particles, i.e

$$\mathbf{a}_i(\mathbf{x}(t)) = \sum_{j \neq i} \mathbf{a}(\|\mathbf{x}_i(t) - \mathbf{x}_j(t)\|). \tag{A.3}$$

Theorem A.1 (Higher-order approximation for isotropic diffusion)**.** *Consider the system described by equation* (A.1)*. Let the particle state* $\mathbf{x}_i(t_k) := \mathbf{x}_{ik}$ *be known for all particles* $i = 1, \ldots, N$*, and let* $\mathbf{A}_{ik} = \mathbf{A}_i(t_k)$*. On the interval* $[t_k, t_{k+1})$*, we have a strong (pathwise) approximation* $\tilde{\mathbf{x}}_i(t)$ *of* $\mathbf{x}_i(t)$ *given by*

$$\begin{aligned}
\tilde{\mathbf{x}}_i(t) &\sim \mathcal{N}(\mathbf{m}_{ik}(t), \mathbf{S}_{ik}(t)), \\
\mathbf{m}_{ik}(t) &= \mathbf{x}_{ik} + \mathbf{a}_{ik}(t - t_k), \\
\mathbf{S}_{ik}(t) &= \mathbf{S}_{1ik}^T(t)\mathbf{S}_{1ik}(t) + \mathbf{S}_{2ik}^T(t)\mathbf{S}_{2ik}(t), \\
\mathbf{S}_{1ik}(t) &= \sigma_i \sqrt{t - t_k}(\mathbf{I} + \frac{t - t_k}{2}\mathbf{A}_{ik}), \qquad \mathbf{S}_{2ik}(t) = \sigma_i \frac{(t - t_k)^{\frac{3}{2}}}{\sqrt{12}}\mathbf{A}_{ik}.
\end{aligned} \tag{A.4}$$

[*]Chalmers tvärgata 3, 412 58 Gothenburg, Sweden, guslindw@chalmers.se
[†]Chalmers tvärgata 3, 412 58 Gothenburg, Sweden, gerlee@chalmers.se

B Improvement of convergence compared to Euler-Maruyama

In this section, we derive an approximation of how much the weak convergence improves as a result of the extra steps taken in Theorem A.1. We will need to define both the Kolmogorov backward equation and Feynman-Kac formula for this proof. We will also make use of the mean-field approximation for the N-particle distribution.

Definition B.1 (Mean-field approximation of particle density)**.** *Consider a stochastic process* $\mathbf{x}(t)$ *described by* (A.1)*, and denote by* $P_k(\mathbf{x}, t)$ *the joint distribution of all particle locations at time* t *conditioned on observing them at time* t_k*. The mean-field approximation of* $P_k(\mathbf{x}, t)$ *is given as [1]*

$$P_k(\mathbf{x}, t) = \prod_{i=1}^{N} p_{ik}(\mathbf{x}, t)$$

where p_{ik} *is the distribution for the i:th particle. This approximation of the* N*-particle distribution results in the highest possible entropy [2].*

Definition B.2 (Kolmogorov backward equation with mean-field closure and its solution via the Feynman-Kac formula.)**.** *The mean field Kolmogorov backward equation for a single particle* i *evolving according to* (A.1) *on a time interval* $[t_k, t_{k+1}]$ *is given by [3]*

$$\begin{aligned} \partial_t p_{ik}(\mathbf{x}, t) &= -\frac{b_i^2}{2} \Delta p_{ik}(\mathbf{x}, t) - (N-1) \nabla p_{ik}^T(\mathbf{x}, t) \{\mathbf{a} * p_{ik}\}(\mathbf{x}, t), \\ p_{ik}(\mathbf{x}, t_{k+1}) &= \varphi(\mathbf{x}). \end{aligned} \tag{B.1}$$

where φ *is a smooth test function with compact support, and* $\{\mathbf{a} * p\}(\mathbf{x}, t) = \int_{\mathbf{R}^2} \mathbf{a}(\mathbf{x} - \mathbf{y}) p(\mathbf{y}, t) \mathrm{d}\mathbf{y}$*, defined element-wise for the vector-valued function* $\mathbf{a}$*. Its solution is given by the Feynman-Kac formula*

$$p_{ik}(\mathbf{x}, t) = \mathbf{E}[\varphi(\mathbf{x}(t_{k+1})) | \mathbf{x}(t) = \mathbf{x}]. \tag{B.2}$$

Theorem B.1 (Weak convergence improvement of the higher-order approximation)**.** *Consider a time interval* $[t_k, t_{k+1})$*, a stochastic process* $\mathbf{x}(t)$ *described by* (A.1) *and an approximate solution* $\tilde{\mathbf{x}}(t)$ *given by* (A.4) *with symmetric* $\mathbf{A}_{ik}$*. Let* $\varphi(\mathbf{x})$ *be a test function with a sufficient number of bounded derivatives. Given a deterministic initial state* $\mathbf{x}(t_k) = \tilde{\mathbf{x}}(t_k) = \mathbf{x}_k$ *we have the following weak convergence;*

$$|\mathbf{E}[\varphi(\tilde{\mathbf{x}}(t_{k+1}))] - \mathbf{E}[\varphi(\mathbf{x}(t_{k+1}))]| \leq C_H (t_{k+1} - t_k). \tag{B.3}$$

Furthermore, under the stability assumption that $\{\mathbf{a} * p\}$ *is Lipschitz continuous with respect to time and enjoys second-order smooth derivatives, we get for all* $i = 1, \ldots, N$ *a sufficient but not necessary bound of*

$$b_i (t_{k+1} - t_k) \Big(\frac{4}{3} (\|\mathbf{A}_{ik}\|_\infty + \frac{3}{4})^2 + \frac{5}{4} \Big) < 2(1 + C_Q) \tag{B.4}$$

leading to a smaller C_H *than the corresponding* C_{EM} *for the Euler-Maruyama method. Here,* C_Q *is a constant dependent on the regularity of the problem and the Euler-Maruyama constant.*

Proof. Assuming the number of particles $N \gg 1$ and utilizing the mean field approximation (Definition 3.2), we realize that we can carry out the proof on a particle-by-particle basis. Thus, we hereby drop the subscript ik from p_{ik} as we are always on the same time interval, and the particles are exchangeable in the mean field setting. We begin by noting that since all $\mathbf{x}_{ik}$ are known, we have that

$$p(\mathbf{x}_{ik}, t_k) = \mathbf{E}[\varphi(\mathbf{x}(t_{k+1}))]. \tag{B.5}$$

Writing out the numerical approximation to solve (A.1) using (A.4), we have

$$\tilde{\mathbf{x}}_i(t) = \mathbf{x}_{ik} + \mathbf{a}_{ik}(t - t_k) + \sigma_i \underbrace{\int_{t_k}^{t} \mathrm{d}W_s}_{:=Z_1(t)} + \sigma_i \mathbf{A}_{ik} \underbrace{\int_{t_k}^{t} W_s \mathrm{d}s}_{:=Z_2(t)}. \tag{B.6}$$

We decompose the random variables $Z_1(t)$ and $Z_2(t)$ as

$$Z_1(t) = \sqrt{t - t_k} U_1 \tag{B.7}$$

$$Z_2(t) = \frac{(t - t_k)^{\frac{3}{2}}}{2} \big(U_1 + \frac{1}{\sqrt{3}} U_2\big), \tag{B.8}$$

where U_1, U_2 are standard normally distributed random variables. Hence, we can get through Itô's lemma that

$$\begin{aligned} p(\tilde{\mathbf{x}}(t_{k+1}), t_{k+1}) - p(\tilde{\mathbf{x}}(t_k), t_k) = &\int_{t_k}^{t_{k+1}} \big(\partial_t p + (\nabla p)^T \mathbf{a}_{ik}\big) \mathrm{d}t \qquad \text{(B.9)} \\ &+ \int_{t_k}^{t_{k+1}} \frac{b_i^2}{2} \mathrm{Tr}\big([\mathbf{I} + \frac{t - t_k}{2} \mathbf{A}_{ik}]^T \nabla^2 p [\mathbf{I} + \frac{t - t_k}{2} \mathbf{A}_{ik}] + \frac{(t - t_k)^2}{12} \mathbf{A}_{ik}^T \nabla^2 p \mathbf{A}_{ik}\big) \mathrm{d}t \\ &+ \int_{t_k}^{t_{k+1}} \nabla p \mathbf{M}_{ik} \mathrm{d}W_t \end{aligned}$$

where $p = p(\tilde{\mathbf{x}}(t), t)$ and $\nabla^2 p$ is its Hessian matrix. As we know that the Itô integral on the third row is a martingale, we simply sum its complicated terms into a single $\mathbf{M}_{ik}$; it will be eliminated the moment we take expectation. We continue by plugging $\tilde{\mathbf{x}}(t)$ into (B.1) for the equality

$$\partial_t p(\tilde{\mathbf{x}}(t), t) = -\frac{b_i^2}{2} \Delta p(\tilde{\mathbf{x}}(t), t) - (N - 1) \nabla p(\tilde{\mathbf{x}}(t), t)^T \{\mathbf{a} * p\}(\tilde{\mathbf{x}}(t), t) \tag{B.10}$$

and plug (B.10) into (B.9) to get

$$\begin{aligned} p(\tilde{\mathbf{x}}(t_{k+1}), t_{k+1}) - p(\tilde{\mathbf{x}}(t_k), t_k) = &\int_{t_k}^{t_{k+1}} (\nabla p)^T (\mathbf{a}_{ik} - (N - 1) \mathbf{a} * p) \mathrm{d}t \qquad \text{(B.11)} \\ &+ \int_{t_k}^{t_{k+1}} \underbrace{\frac{b_i^2}{2} \mathrm{Tr}\big([\mathbf{I} + \frac{t - t_k}{2} \mathbf{A}_{ik}]^T \nabla^2 p [\mathbf{I} + \frac{t - t_k}{2} \mathbf{A}_{ik}] + \frac{(t - t_k)^2}{12} \mathbf{A}_{ik}^T \nabla^2 p \mathbf{A}_{ik} - \nabla^2 p\big)}_{:=D_{ik}(p(\tilde{\mathbf{x}}(t), t))} \mathrm{d}t \\ &+ \int_{t_k}^{t_{k+1}} \nabla p \mathbf{M}_{ik} \mathrm{d}W_t \end{aligned}$$

where D_{ik} is a linear, time-dependent, second order differential operator.
Next we note that $p(\tilde{\mathbf{x}}(t_k), t_k) = \mathbf{E}[\varphi(\mathbf{x}(t_{k+1}))]$ just as in (B.5). Now we are ready to take the expected value of (B.11);

$$\begin{aligned}\mathbf{E}[\varphi(\tilde{\mathbf{x}}(t_{k+1}))] - \mathbf{E}[\varphi(\mathbf{x}(t_{k+1}))] = &\int_{t_k}^{t_{k+1}} \mathbf{E}[(\nabla p(\tilde{\mathbf{x}}(t), t))^T(\mathbf{a}_{ik} - (N-1)\mathbf{a} * p)]\mathrm{d}t \\ &+ \int_{t_k}^{t_{k+1}} \frac{b_i^2}{2}\mathbf{E}\big[D_{ik}(p(\tilde{\mathbf{x}}(t), t))\big]\mathrm{d}t\end{aligned} \tag{B.12}$$

Note that the left-hand side of (B.12) is the quantity which we wish to find a bound for. With this, we have arrived at the point where we would be well served a reminder that the calculations we have carried out are almost the exact same as the standard proof of weak convergence for the Euler-Maruyama scheme. In fact, by setting $\mathbf{A}_{ik} = \mathbf{0}$, we get the Euler-Maruyama scheme, and as such we can use the standard arguments based on the triangle inequality to prove weak convergence. However, we are at this moment more interested in under what circumstances the additional terms *improve* said weak convergence.

We note that the matrices $\mathbf{S}_{1ik}(t)$ and $\mathbf{S}_{2ik}(t)$ are symmetric if $\mathbf{A}_{ik}$ is symmetric. After noting this, we make use of the property that the trace of a product of matrices is invariant under any permutation of said symmetric matrices. Thus, we can conclude that

$$D_{ik}(t) = \frac{1}{2}\mathrm{Tr}\big((\mathbf{S}_{1ik}^T(t)\mathbf{S}_{1ik}(t) + \mathbf{S}_{2ik}^T(t)\mathbf{S}_{2ik}(t) - \frac{b_i^2}{2}\mathbf{I})\nabla^2 p(x,t)\big). \tag{B.13}$$

Remember that we have derived an exact expression for the eigenvalues of $\mathbf{S}_{ik}(t)$ with the largest being

$$\lambda_M = \frac{\mathrm{Tr}(\mathbf{S}_{ik}) + \sqrt{\mathrm{Tr}(\mathbf{S}_{ik})^2 - 4|\mathbf{S}_{ik}|}}{2}. \tag{B.14}$$

The approach now will be to bound $\mathbf{S}_{ik} := \mathbf{S}_{ik}(t_{k+1})$ with an *isotropic* diffusion matrix, i.e an identity matrix multiplied with the principal eigenvalue of $\mathbf{S}_{ik}$, i.e

$$D_{ik}(t) \leq \underbrace{\frac{\mathrm{Tr}(\mathbf{S}_{ik}) + \sqrt{\mathrm{Tr}(\mathbf{S}_{ik})^2 - 4|\mathbf{S}_{ik}|} - 2b_i^2}{4}}_{:=\beta_{ik}}\left(\frac{\partial^2}{\partial x_1^2} + \frac{\partial^2}{\partial x_2^2}\right) \tag{B.15}$$

Using this maximum we get that

$$\begin{aligned}\big|\mathbf{E}[\varphi(\tilde{\mathbf{x}}(t_{k+1}))] - \mathbf{E}[\varphi(\mathbf{x}(t_{k+1}))]\big| \leq \Big| &\int_{t_k}^{t_{k+1}} \mathbf{E}[(\nabla p(\tilde{\mathbf{x}}(t), t))^T](\mathbf{a}_{ik} - (N-1)\mathbf{a} * p) \\ &+ \beta_{ik}\mathbf{E}[\Delta p(\tilde{\mathbf{x}}(t), t)]\mathrm{d}t\Big|\end{aligned} \tag{B.16}$$

which given bounded derivatives ∇p, $\partial^2 p/(\partial x_j \partial x_l)$ proves the weak convergence (for details, see [4]). Next, we see that to improve on the bound given by the Euler-Maruyama scheme,

we must have that

$$\Big|\underbrace{\int_{t_k}^{t_{k+1}} \mathbf{E}_{\overline{p}}[(\nabla p^T)(\mathbf{a}_{ik}-(N-1)\mathbf{a}*p)]\mathrm{d}t}_{Q_1}+\beta_{ik}\underbrace{\int_{t_k}^{t_{k+1}} \mathbf{E}_{\overline{p}}[\Delta p]\mathrm{d}t}_{Q_2}\Big|< \Big|\underbrace{\int_{t_k}^{t_{k+1}} \mathbf{E}_{\hat{p}}[(\nabla p)^T)(\mathbf{a}_{ik}-(N-1)\mathbf{a}*p)]\mathrm{d}t}_{Q_3}\Big| \tag{B.17}$$

where we now use the subscripts $\hat{p}$ and $\overline{p}$ to denote expectation with respect to the density generated by the Euler-Maruyama scheme and the higher order scheme respectively. Conditions for when (B.17) holds depends on the sign of Q_1 and Q_2, and most importantly if they are of the same or opposite sign. We start with the case of Q_1 and Q_2 having opposite signs; we get by the reverse triangle inequality that

$$||Q_1|-\beta_{ik}|Q_2||<|Q_3|\rightarrow \beta_{ik}<\frac{|Q_1|+|Q_3|}{|Q_2|} \tag{B.18}$$

In the case of Q_1 and Q_2 having the same signs, we must instead have that

$$\beta_{ik}<\frac{|Q_3|-|Q_1|}{|Q_2|}. \tag{B.19}$$

We know from weak convergence of the Euler-scheme that $|Q_3|<C_{EM}(t_{k+1}-t_k)$, and from regularity of solutions to (B.1) that Q_1 and Q_2 exists. Setting $(|Q_3|-|Q_1|)/|Q_2|:=C_Q$, we have

$$\beta_{ik}<\frac{1}{2}b_i(t_{k+1}-t_k)\big(\frac{4}{3}(\|\mathbf{A}_{ik}\|_\infty+\frac{3}{4})^2+\frac{5}{4}\big)-1\Rightarrow$$
$$b_i(t_{k+1}-t_k)\big(\frac{4}{3}(\|\mathbf{A}_{ik}\|_\infty+\frac{3}{4})^2+\frac{5}{4}\big)<2(1+C_Q). \tag{B.20}$$

Thus, as long as (B.20) is satisfied, (A.4) enjoys superior convergence compared to the Euler-Maruyama scheme. □

C Psuedo code for implementation

We chose to model our cell population using a system of interacting stochastic differential equations with isotropic diffusion. At a particular moment in time t, the system evolves according to the following set of equations

$$\mathrm{d}\mathbf{x}_i=-\nabla V(\mathbf{x}_i,t)\mathrm{d}t+\Sigma_i\mathrm{d}W(t), \tag{C.1}$$

$$V(\mathbf{x},t)=\sum_{j=1}^{N_t}U(\|\mathbf{x}-\mathbf{x}_j\|), \tag{C.2}$$

$$U(r)=D_e\Big[\Big(\frac{\varphi(r)}{\varphi(r_0)}\Big)^{2a}-2\Big(\frac{\varphi(r)}{\varphi(r_0)}\Big)^{a}\Big]. \tag{C.3}$$

Algorithm 1: Bayesian inference on σ_i in the model given by (C.1)

Input:
•A data set consisting of K observations of N_k particles observed at time t_k, $k = 0, \ldots, K$. ;
•An interaction potential $\varphi(\mathbf{x})$.;
•A prior distribution Gamma(α_0, β_0).;
•A list of indices ν for what other particles are within the interaction range of particle i, $i = 1, \ldots, N$.;
$\alpha_K = [0, 0 \ldots, 0]$, N zeros.;
$\beta_K = [0, 0 \ldots, 0]$, N zeros.;
for $k = 0 : K - 1$ **do**
    for $i = 1 : N$ **do**
        Calculate $\mathbf{m}_{ik} = \mathbf{x}_{ik} + \mathbf{a}_{ik}(t_{k+1} - t_k)$;
        Calculate $\overline{\mathbf{S}}_{1ik} = \sqrt{t_{k+1} - t_k}(\mathbf{I} + \frac{t_{k+1} - t_k}{2}\mathbf{A}_{ik})$;
        Calculate $\overline{\mathbf{S}}_{2ik} = \frac{(t_{k+1} - t_k)^{3/2}}{\sqrt{12}}\mathbf{A}_{ik}$;
        Calculate $\overline{\mathbf{S}}_{ik} = \overline{\mathbf{S}}_{1ik}^T \overline{\mathbf{S}}_{1ik} + \overline{\mathbf{S}}_{2ik}^T \overline{\mathbf{S}}_{2ik}$;
        $\alpha[i] \leftarrow \alpha[i] + 1$;
        $\beta[i] \leftarrow \beta[i] + \frac{1}{2}\big(\mathbf{x}_{i(k+1)} - \mathbf{m}_k\big)^T \overline{\mathbf{S}_k}^{-1} \big(\mathbf{x}_{i(k+1)} - \mathbf{m}_k\big)$

Algorithm 2: Calculate $\mathbf{a}_{ik}$

Input:
•A data set consisting of N_k particles observed at time t_k. ;
•An interaction potential $\varphi(\mathbf{x})$.;
•An index i for the cell we wish to calculate $\mathbf{a}_{ik}$ for.;
•A list of indices ν for what other particles are within the interaction range of particle i.;
$\mathbf{a}_{ik} = [0, 0]^T$;
for $j = 1 : N$ **do**
    if $j \in \nu$ **then**
        $\mathbf{a}_{ik} \leftarrow \mathbf{a}_{ik} + \nabla_i \varphi(\mathbf{x}_{ik} - \mathbf{x}_{jk})$;
return $\mathbf{a}_{ik}$

Algorithm 3: Calculate $\nabla_i \varphi(\mathbf{x}_{ik} - \mathbf{x}_{jk})$

```
Input:
•A 'me' particle position x_ik and a 'you' particle position x_jk time t_k. ;
•An interaction potential φ(x).;
•A difference Δx.;
```

$$
\begin{aligned}
\mathbf{x}_{+1} &= \mathbf{x}_{ik} + \frac{1}{2}\Delta x \begin{bmatrix} 1 \\ 0 \end{bmatrix} \\
\mathbf{x}_{-1} &= \mathbf{x}_{ik} - \frac{1}{2}\Delta x \begin{bmatrix} 1 \\ 0 \end{bmatrix} \\
\mathbf{x}_{+2} &= \mathbf{x}_{ik} + \frac{1}{2}\Delta x \begin{bmatrix} 0 \\ 1 \end{bmatrix} \\
\mathbf{x}_{-2} &= \mathbf{x}_{ik} - \frac{1}{2}\Delta x \begin{bmatrix} 0 \\ 1 \end{bmatrix} \\
a_1 &= \frac{1}{\Delta x}\big(\varphi(\mathbf{x}_{+1} - \mathbf{x}_{jk}) - \varphi(\mathbf{x}_{-1} - \mathbf{x}_{jk})\big) \\
a_2 &= \frac{1}{\Delta x}\big(\varphi(\mathbf{x}_{+2} - \mathbf{x}_{jk}) - \varphi(\mathbf{x}_{-2} - \mathbf{x}_{jk})\big) \\
\nabla_i \varphi &= \begin{bmatrix} a_1 \\ a_2 \end{bmatrix}
\end{aligned}
$$

return $\nabla_i \varphi$

Algorithm 4: Calculate $\mathbf{A}_{ik}$

```
Input:
•A data set consisting of N_k particles observed at time t_k. ;
•An interaction potential φ(x).;
•An index i for the cell we wish to calculate A_ik for.;
•A list of indices ν for what other particles are within the interaction range of
 particle i.;
a_ik = [0, 0]^T;
for j = 1 : N do
    if j ∈ ν then
        A_ik ← A_ik + Hessian_i(φ(x_ik − x_jk));
return A_ik
```

Algorithm 5: Calculate $\text{Hessian}_i(\varphi(\mathbf{x}_{ik} - \mathbf{x}_{jk}))$

Input:

•A 'me' particle position $\mathbf{x}_{ik}$ and a 'you' particle position $\mathbf{x}_{jk}$ time t_k. ;

•An interaction potential $\varphi(\mathbf{x})$.;

•A difference Δx.;

$$
\begin{aligned}
\mathbf{x}_{+1} &= \mathbf{x}_{ik} + \frac{1}{2}\Delta x \begin{bmatrix} 1 \\ 0 \end{bmatrix} \\
\mathbf{x}_{-1} &= \mathbf{x}_{ik} - \frac{1}{2}\Delta x \begin{bmatrix} 1 \\ 0 \end{bmatrix} \\
\mathbf{x}_{+2} &= \mathbf{x}_{ik} + \frac{1}{2}\Delta x \begin{bmatrix} 0 \\ 1 \end{bmatrix} \\
\mathbf{x}_{-2} &= \mathbf{x}_{ik} - \frac{1}{2}\Delta x \begin{bmatrix} 0 \\ 1 \end{bmatrix} \\
a_{11} &= \varphi(\mathbf{x}_{+1} - \mathbf{x}_{jk1}) - 2\varphi(\mathbf{x}_{ik1} - \mathbf{x}_{jk1}) + \varphi(\mathbf{x}_{-1} - \mathbf{x}_{jk1}) \\
a_{22} &= \varphi(\mathbf{x}_{+2} - \mathbf{x}_{jk2}) - 2\varphi(\mathbf{x}_{ik2} - \mathbf{x}_{jk2}) + \varphi(\mathbf{x}_{-2} - \mathbf{x}_{jk2}) \\
a_{12} &= \varphi\big(\frac{\mathbf{x}_{+1} + \mathbf{x}_{+2}}{2} - \mathbf{x}_{jk}\big) - \varphi\big(\frac{\mathbf{x}_{+1} + \mathbf{x}_{-2}}{2} - \mathbf{x}_{jk}\big) \\
&\quad - \varphi\big(\frac{\mathbf{x}_{-1} + \mathbf{x}_{+2}}{2} - \mathbf{x}_{jk}\big) + \varphi\big(\frac{\mathbf{x}_{-1} + \mathbf{x}_{-2}}{2} - \mathbf{x}_{jk}\big)
\end{aligned}
$$

return $\text{Hessian}_i(\varphi) = \frac{1}{\Delta x^2} \begin{bmatrix} a_{11} & a_{12} \\ a_{12} & a_{22} \end{bmatrix}$

References

[1] Bruna M, Chapman SJ, Robinson M. Diffusion of particles with short-range interactions. SIAM Journal on Applied Mathematics. 2017;77(6):2294-316.

[2] Singer A. Maximum entropy formulation of the Kirkwood superposition approximation. The Journal of chemical physics. 2004;121(8):3657-66.

[3] Klebaner FC. Introduction to stochastic calculus with applications. World Scientific Publishing Company; 2012.

[4] Carlsson J, Moon KS, Szepessy A, Tempone R, Zouraris G. Stochastic Differential Equations: Models and Numerics. Lecture notes. 2019.
